# Recent Advances in Antimicrobial Resistance: Insights from *Escherichia coli* as a Model Organism

**DOI:** 10.3390/microorganisms13010051

**Published:** 2024-12-31

**Authors:** Zhaoyang Zhang, Minliang Wei, Bin Jia, Yingjin Yuan

**Affiliations:** Frontier Science Center for Synthetic Biology and Key Laboratory of Systems Bioengineering (Ministry of Education), School of Chemical Engineering and Technology, Tianjin University, Tianjin 300072, China; zhaoyangz@tju.edu.cn (Z.Z.); mlwei@tju.edu.cn (M.W.); yjyuan@tju.edu.cn (Y.Y.)

**Keywords:** antimicrobial resistance, machine learning, *Escherichia coli*, synthetic biology, AMR prevention

## Abstract

Antimicrobial resistance (AMR) represents a critical global health threat, and a thorough understanding of resistance mechanisms in *Escherichia coli* is needed to guide effective treatment interventions. This review explores recent advances for investigating AMR in *E. coli*, including machine learning for resistance pattern analysis, laboratory evolution to generate resistant mutants, mutant library construction, and genome sequencing for in-depth characterization. Key resistance mechanisms are discussed, including drug inactivation, target modification, altered transport, and metabolic adaptation. Additionally, we highlight strategies to mitigate the spread of AMR, such as dynamic resistance monitoring, innovative therapies like phage therapy and CRISPR-Cas technology, and tighter regulation of antibiotic use in animal production systems. This review provides actionable insights into *E. coli* resistance mechanisms and identifies promising directions for future antibiotic development and AMR management.

## 1. Introduction

Antimicrobial resistance (AMR) significantly impacts human health, exacerbated by the overuse of antibiotics, leading to a rise in drug-resistant bacteria. According to Antimicrobial Resistance Collaborators, bacterial infections were the second leading cause of death globally in 2019, following ischemic heart disease, with approximately 4.95 million deaths attributed to bacterial AMR [1]. Fluoroquinolones and beta-lactam antibiotics are commonly used as first-line treatments for severe infections, but resistance to these antibiotics contributes to over 70% of AMR-related deaths. The highest fatal burden of antibiotic resistance comes from bloodstream infections, followed by intra-abdominal and respiratory infections. Resistance in *Escherichia coli* can lead to serious diseases such as sepsis and meningitis, threatening human health [2]. A 2010 study reported that *E. coli* pyogenic infections emerged as a new infectious disease among patients with hematological malignancies [3]. All patients had underlying blood malignancies and were initially treated with fluoroquinolones, but the *E. coli* strains were resistant, leading to treatment failure and two deaths. Furthermore, resistant *E. coli* spreads in food, water, and the environment, directly or indirectly impacting human, animal, and environmental health [4]. For instance, Shiga toxin-producing *E. coli* (STEC) is a zoonotic pathogen that can be transmitted to humans through contaminated food, water, or contact with infected environments or animals, causing gastrointestinal complications [5]. Research on *E. coli* drug resistance is crucial for understanding its mechanisms and improving drug administration and development. For example, ampicillin, ciprofloxacin, and gentamicin are strongly metabolism-dependent antibiotics, whereas colistin and mitomycin C are weakly metabolism-dependent. Combining these types of antibiotics can effectively target both active and metabolically resistant *E. coli* strains, optimizing antibiotic use [6]. Screening of antibiotic-sensitive mutant libraries can help us understand the genetic basis of intrinsic bacterial resistance, study the activity profile of antibiotics, and then improve or develop new drugs against pathogenic bacteria.

## 2. Methods for Studying Resistance in *E. coli*

Researchers have employed a variety of methods to study antibiotic resistance, including machine learning, laboratory evolution, the construction of mutant libraries, and whole-genome sequencing. Each of these approaches contributes to the understanding of antibiotic resistance in bacteria.

### 2.1. Machine Learning in Resistance Pattern Analysis

Clinical datasets are underutilized for tracking the scale of antibiotic resistance. Researchers sought patterns of resistance in the Antimicrobial Testing Leadership and Surveillance (ATLAS) database to study the development of minimum inhibitory concentrations (MIC) for pathogens isolated from patients over the past decade. ATLAS was also used to predict changes in drug resistance, aiding in forecasting which pathogens might develop clinical resistance in the future [7].

**Figure 1:**
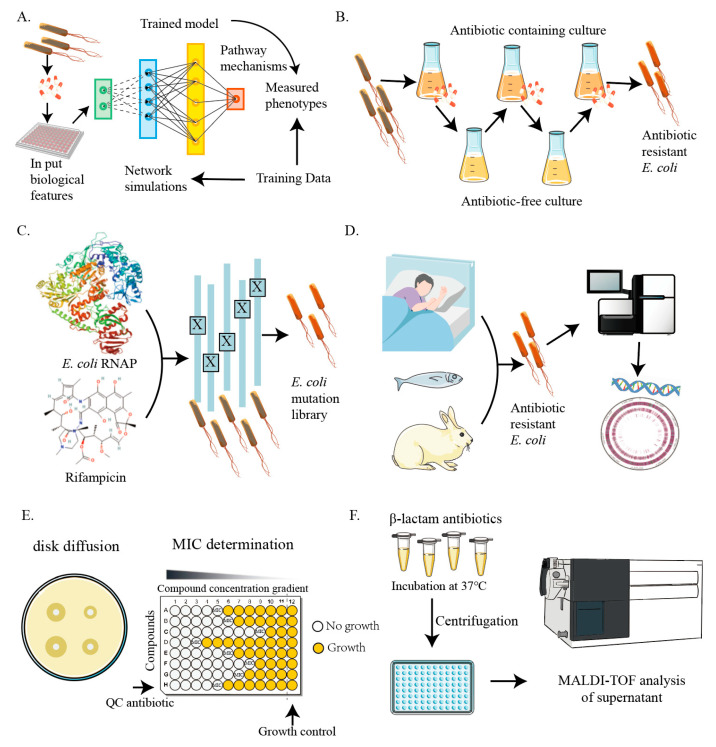
Illustration of research methods for studying *E. coli* antibiotic resistance. (**A**) Exploration of resistance mechanisms using machine learning. (**B**) Investigation of resistant strains obtained through laboratory evolution. (**C**) Analysis of resistance genes and mechanisms through the construction of mutant libraries. (**D**) Analysis of resistance genes using whole-genome sequencing. (**E**) Traditional disk diffusion and MIC methods for antibiotic resistance testing. (**F**) Detection of β-lactamase-producing bacteria using MALDI-TOF MS.

A network model-driven machine learning approach can overcome the limitations of traditional “black-box” methods when analyzing large biological datasets [8]. This approach reveals how antibiotics affect bacterial death through metabolic pathways. In the model, input conditions (such as metabolites) are perturbed, followed by network modeling (such as metabolic flux). These data were then transformed into rich biological network states to train predictive machine learning models, expounding the mechanisms behind antibiotic lethality measurements (Figure 1A).

The machine learning model revealed that central metabolic pathways have similar effects on ampicillin, ciprofloxacin, and gentamicin, while the purine biosynthesis pathway uniquely affects gentamicin. Mutations in the purine biosynthesis pathway—such as deletions of *purD* (glycinamide ribonucleotide synthase), *purE* (N5-carboxyaminoimidazole ribonucleotide isomerase), *purK* (5-(carboxyamino)imidazole ribonucleotide synthase), or *purM* (phosphoribosylformylglycinamidine cyclase)—reduce the lethality of ampicillin and ciprofloxacin against *E. coli* but increase the lethality of gentamicin against *E. coli*. Additionally, adenine limitation induced by antibiotics increases the cellular demand for ATP, enhancing respiratory and metabolic activity, which in turn improved the bactericidal effects of the antibiotics [9,10]. Antibiotics disrupt cellular processes, impacting the entire nucleotide pool, including adenine. A decrease in adenine levels compels bacteria to enhance purine biosynthesis to maintain nucleotide supply. This increased demand exerts stress on metabolic pathways, resulting in elevated ATP requirements. To compensate for adenine limitations, bacteria accelerate central carbon metabolism to meet energy requirements. This intensification of metabolic activity leads to heightened respiration rates and increased oxygen consumption. Although a high metabolic state facilitates ATP production, essential for cellular functions, it can also expedite cell death in the presence of antibiotics. The accumulation of toxic byproducts, such as reactive oxygen species (ROS), may contribute to bacterial cell death. Additionally, alterations in metabolic activity can disrupt cellular homeostasis, making bacteria more susceptible to antibiotic-mediated damage [11,12,13].

### 2.2. Laboratory Evolution and Resistant Mutants

In traditional studies, *E. coli* was passaged daily into fresh antibiotic-containing media to promote strain persistence and enrich those that acquired resistance mutations. Through continuous laboratory evolution, strains with mutations in the *nuo* operon were identified. These mutations disrupted the proton pump, increased cytoplasmic acidification, and conferred resistance to antibiotics [14]. Cytoplasmic acidification can drive bacteria into a persister state, significantly increasing their tolerance to antibiotics. In this state, bacterial growth and metabolic activity are markedly reduced, which diminishes the efficacy of antibiotic treatments. This induction of the persister state is thought to occur through two primary mechanisms: first, acidification activates the RpoS regulon, enhancing bacterial resistance to environmental stresses and potentially leading to the upregulation of resistance genes. Second, under more severe cytoplasmic acidification, protein synthesis is shut down, further reducing the impact of antibiotics, as many of these drugs rely on normal protein synthesis for their antimicrobial effects [14].

The traditional approach to laboratory evolution involves cultivating bacterial strains in media supplemented with antibiotics and then screening for resistant mutants by increasing the antibiotic concentration daily. However, constant antibiotic exposure can lead to growth-dependent strains, hindering the enrichment of lower-frequency resistance mutations. In contrast, another group of researchers employed an intermittent administration method to screen *E. coli* mutants resistant to carbenicillin, streptomycin, and ciprofloxacin (Figure 1B). This approach led to the discovery of resistant mutants associated with core metabolism [15].

In the intermittent evolution method, *E. coli* was cultured daily at 37 °C for 22 h and then maintained at a specific temperature for 30 min to allow transcriptional adaptation. The strain was then exposed to the antibiotic at the same temperature for 1 h, washed twice with phosphate-buffered saline (PBS), diluted 500-fold, and reinoculated into antibiotic-free media. This process was repeated over 10 days, with the incubation temperature rising by 1 °C each day, starting from 20 °C and reaching 30 °C on the final day. This intermittent evolution approach uncovered both classical resistance genes, such as *ompF*, *acrD*, and *gyrA*, as well as metabolic genes like *sucA*, *gltD*, *ushA*, *icd*, *ycgG*, and *yidA*. Phenotypic and genotypic analysis revealed that wild-type *E. coli* exhibited significantly increased metabolic activity after carbenicillin treatment, while the central metabolic and energy activity in the 2-oxoglutarate dehydrogenase (*sucA*) mutant (*sucA^M^*) was generally downregulated. In wild-type *E. coli*, exposure to carbenicillin induces an increase in central carbon metabolism pathways. This upregulation enhances the bacteria’s metabolic activity and energy production, thereby facilitating survival under antibiotic stress. The increased metabolic activities observed after carbenicillin treatment can be attributed to several factors. Bacteria may initiate stress responses that enhance metabolic processes as they adapt to antibiotic challenges. This can lead to the upregulation of specific pathways critical for survival, allowing the bacteria to counteract the inhibitory effects of carbenicillin. Additionally, the downregulation of certain pathways may redirect metabolic resources toward those that enhance resistance and survival under antibiotic stress. It is hypothesized that resistance in *sucA^M^* may arise by bypassing carbenicillin-mediated activation of the tricarboxylic acid cycle, thereby preventing metabolic toxicity and cell death. When the addition of antibiotics disrupts cellular metabolism, it leads to metabolic toxicity, which refers to the harmful effects on a cell’s imbalanced metabolic processes that can lead to cellular dysfunction or death. It often arises when metabolic pathways become overwhelmed or disrupted [15].

In the context of antibiotics, metabolic toxicity can occur when the drug disrupts normal cellular metabolism. For example, certain antibiotics can inhibit essential enzymes in metabolic pathways, leading to an accumulation of toxic metabolites or a depletion of critical cellular energy sources like ATP. This disruption can overwhelm the cell’s ability to maintain homeostasis, resulting in a state of metabolic imbalance. The connection between metabolic toxicity and cell death lies in the fact that severe disruptions to metabolism can compromise vital cellular functions, such as energy production, biosynthesis, and repair mechanisms. When a cell can no longer sustain these functions, it may enter a state of stress that ultimately leads to apoptosis (programmed cell death) or necrosis (uncontrolled cell death). Thus, while antibiotics aim to kill bacteria by targeting essential functions, they can also indirectly induce cell death through metabolic toxicity by pushing cellular processes beyond their limits [10,16]. Therefore, by bypassing the activation of the tricarboxylic acid cycle, *sucA^M^* may effectively mitigate the adverse effects associated with metabolic toxicity, thereby enhancing bacterial survival under antibiotic pressure.

### 2.3. Mutant Libraries for Mechanistic Insights

Constructing single-gene mutants helps to study the impact of individual genes on *E. coli* antibiotic resistance. In *E. coli* K-12, a single-gene knockout library was built by deleting non-essential gene coding regions, resulting in 3985 mutants from 4288 target genes [17]. Nearly 4000 single-gene knockout mutants from the KEIO collection were further utilized to study antibiotic resistance (Figure 1C). The study found that 283 strains exhibited resistance to at least one of the fourteen antibiotics used, which included ciprofloxacin, rifampicin, vancomycin, ampicillin, sulfamethoxazole, gentamicin, metronidazole, streptomycin, fusidic acid, tetracycline, chloramphenicol, furantoin, erythromycin, and triclosan. These 283 strains were further tested against eight additional antibiotics—pomomycin, cephaloadine, amtraxam, colistin, neomycin, enoxacin, tobramycin, and cefoxitin—to assess their antibiotic sensitivity [18]. This study revealed the resistance characteristics of the inherent genes of *E. coli* and provided a valuable target for antibiotic drug design.

The Random Genome Mutation-directed Evolution (DIvERGE) method can generate a comprehensive mutagenic library [19]. This approach involves overlaying long genome segments with successive mutant oligos containing overlapping sequences, which are randomly generated by the algorithm. This design enables the creation of a mutagenic library with a long-range and uniform distribution of mutations. Using the DIvERGE method, multiple mutations in the *folA* gene were obtained, resulting in high-level resistance to trimethoprim. Through five successive rounds of DIvERGE on *folA*, resistance was increased 895-fold compared to the wild type. The method also identified resistance mutations to fluoroquinolones, such as ciprofloxacin, at the *gyrA* site. DIvERGE technology can also predict antibiotic resistance during drug development stages in clinical trials [19].

In the study of rifampicin’s binding site on *E. coli*, Multiplex Automated Genome Engineering (MAGE) was used to introduce single amino acid mutations into the rifampicin-binding site of *E. coli* RNA polymerase (RNAP). This targeted approach allows for a detailed investigation of the rifampicin (Rif) binding site within the RNA polymerase (RNAP) complex. By employing multiplex automated genome engineering (MAGE), researchers can systematically introduce single amino acid substitutions at specific positions in the *rpoB* gene, particularly those known to interact with Rif. This method enables a comprehensive understanding of how these mutations affect Rif binding and overall bacterial physiology. Although RNAP is a holoenzyme composed of multiple subunits, the *rpoB* mutations are critical for elucidating mechanisms of antibiotic action and resistance [20,21,22,23,24,25,26,27,28]. By concentrating on *rpoB*, we can characterize a specific region involved in Rif binding, revealing how various substitutions influence drug sensitivity and the functional dynamics of the transcription machinery. This focused mutagenesis provides valuable insights into the complexities of antibiotic resistance and the physiological implications of changes in RNAP activity. Each amino acid residue at the binding site was mutated to one of nineteen possible residues, creating a total of 760 mutants that covered the entire rifampicin-binding site [29]. Genetic maps of drug–enzyme interactions revealed where alpha-helix mutations significantly affect rifampicin binding. In addition, mutations at other binding sites of RNAP altered its activity, which affected nucleotide consumption within the cell. These changes in nucleotide dynamics subsequently influenced the cell’s sensitivity to various antibiotics, potentially increasing or decreasing its resistance depending on the nature of the mutation.

In the process of constructing rapid mutations to study antibiotic resistance, some studies on resistance genes aim to avoid changes to the entire genome. In an orthogonal replication system developed using the highly virulent phage PRD1, the DNA polymerase (O-DNAPs) is orthogonal to the host genome’s DNA polymerase. This allows for selective replication and rapid mutation of inserted fragments without affecting the *E. coli* genome. This method establishes an orthogonal system in *E. coli* that accelerates continuous evolution. By targeting mutations in the tigecycline resistance gene, it increased the resistance of *E. coli* by 150-fold [30].

Another approach to studying mutations that induce antibiotic resistance aims for rapid, comprehensive mutations in the *E. coli* genome to investigate the overall gene network of the strain. To achieve this, an engineered, inducible, self-replicating transposon platform was developed to examine the impact of transposon functions on the evolution of antibiotic resistance phenotypes in *E. coli*. This platform enables continuous, whole-genome mutations and dynamic rewiring of gene networks in bacteria [31].

### 2.4. Genome Sequencing of Resistant Strains

Genome sequencing, especially short-read and long-read sequencing technologies, has become a powerful tool for tracking the evolution and spread of antibiotic resistance genes in *E. coli* [32]. Short-read sequencing, such as Illumina platforms, offers high throughput but is limited by short-read lengths (100–300 bp) [33,34]. This makes it difficult to resolve complex genomic regions, such as repetitive sequences or large structural variants. It is highly effective for detecting small mutations, SNPs, and indels in resistance genes. In contrast, long-read sequencing technologies like PacBio and Oxford Nanopore generate much longer reads (several kilobases) [35,36]. These methods allow for more complete genome assemblies and can identify large structural variants, gene amplifications, and plasmid-based resistance. They are particularly useful for detecting large resistance plasmids and mobile genetic elements [37]. Together, these techniques allow for a more comprehensive understanding of the genetic landscape of antibiotic resistance in *E. coli*, including the identification of resistance-related genes and mobile genetic elements. Through genome sequencing of *E. coli* strains collected from clinical patients and animal samples, researchers are able to track the spread and emergence of antibiotic-resistance genes more effectively (Figure 1D). This approach facilitates better detection and surveillance, helping to understand patterns of resistance and inform regulatory actions.

Researchers also performed whole-genome scanning of *E. coli* strains from wastewater treatment plants, collecting 92 *E. coli* genomes. This large dataset revealed patterns of resistance that may not be apparent in smaller datasets. By using the single recombination breakpoint (SBP) method, recombinant genes were identified and excluded, followed by positive selection analysis of the remaining genes. This analysis revealed eight genes linked to drug resistance, including biofilm formation and changes in membrane permeability associated with bacterial persistence. Notably, mutations linked to resistance were discovered in two genes: the porin gene *ompC* and the persistence gene *hipA*, both through non-synonymous mutations [38]. While the roles of *ompC* and *hipA* mutations in resistance are well-established, this study uncovered new connections between other positively selected genes and resistance mechanisms, such as biofilm formation and efflux pumps. These findings broaden the understanding of resistance pathways. In the complex environment of wastewater treatment plants, *E. coli* faces a variety of stresses, providing insights into how the bacterium adapts to environmental challenges. The genetic variability of *E. coli* genomes in wastewater reveals a broader spectrum of mutations and potential resistance mechanisms, enabling the identification of new genes associated with antibiotic resistance. By associating non-synonymous mutations in positively selected genes with antibiotic resistance profiles, researchers have established clearer connections between specific genetic changes and functional resistance traits. This study reveals new dimensions in the evolution of bacterial antibiotic resistance, highlighting the importance of ecological factors and expanding the knowledge of resistance dynamics.

In addition to the discovery of resistance genes, segmental duplications and gene amplifications have also been identified as contributors to antibiotic resistance [39,40,41,42,43,44,45,46]. In prokaryotes, segment duplications also play a significant role in conferring antibiotic resistance. Mobile genetic elements such as insertion sequences (IS), transposons (Tn), and integrons (In) contribute to structural changes in bacterial genomes, leading to the acquisition of drug resistance [47,48]. Clinical studies have revealed that repeated bacterial genome segments are linked to increased resistance to antibiotics [49,50,51,52,53,54,55,56]. The first case of gene amplification contributing to bacterial antibiotic resistance was discovered in *Salmonella* in 2006 [53]. IS elements can amplify resistance by promoting the overexpression of nearby resistance genes. For example, ISAba1 provides a strong promoter upstream of bla_AmpC_, increasing cephalosporinase production and reducing *Acinetobacter baumannii* and *E. coli* susceptibility to β-lactam antibiotics [57]. Similarly, IS1 insertion upstream of TEM-6 (ultra-broad-spectrum β-lactamase) activates a hybrid promoter, resulting in higher resistance levels in *E. coli* to ceftazidime and aztreonam [58]. In *Staphylococcus aureus*, lateral genetic transfer (LGT) has facilitated the development of multidrug resistance [59]. Further research on *Salmonella* Typhimurium identified gene amplification as a mechanism for increasing resistance [56]. A 23 kb genomic resistance module (GRM) was identified at a chromosomal integration hotspot in *E. coli* from patients with urinary tract infections. This module is responsible for the multidrug resistance observed in *E. coli* [60]. Later, a clinically isolated strain of *E. coli* (907355) was shown to use over 15% of its genome for tandem amplification of ~10 kb resistance modules, allowing it to survive antibiotic treatment [61].

In addition to advanced methods such as genome sequencing, traditional approaches for antibiotic resistance testing in *E. coli* remain critical in both clinical and research settings. These methods, including disk diffusion, minimum inhibitory concentration (MIC) testing, and matrix-assisted laser desorption/ionization time-of-flight (MALDI-TOF) mass spectrometry, continue to play a critical role in routine diagnostics. Disk diffusion is a widely used and simple method where antibiotic-impregnated paper discs are placed on an agar plate inoculated with bacteria. The zone of inhibition around each disc indicates the bacterium’s susceptibility to the antibiotic. This method, while effective for screening large numbers of strains, lacks precision in measuring exact resistance levels and cannot detect resistance mechanisms that do not alter growth inhibition (Figure 1E) [62,63]. MIC testing determines the lowest concentration of an antibiotic required to inhibit bacterial growth. This method is considered the gold standard for assessing the effectiveness of antibiotics against specific strains, providing quantitative data on resistance. However, it is time-consuming and requires precise liquid handling and interpretation (Figure 1E) [64,65]. MALDI-TOF mass spectrometry is a newer technology that has revolutionized microbial identification and resistance profiling. This method utilizes the protein fingerprint of bacterial cells to rapidly identify species and detect resistance markers. MALDI-TOF MS has gained popularity due to its speed, low cost, and ease of use in clinical microbiology labs [66,67]. It facilitates resistance testing by evaluating bacterial growth in media containing antibiotics or isotopic markers, revealing shifts in the spectra that indicate resistance. Specifically, MALDI-TOF MS can detect the modification of antibiotics by resistance determinants, identify resistance-associated clones, and uncover resistance-related proteins such as β-lactamases, which result in the hydrolysis of β-lactam antibiotics, evidenced by the reduction of antibiotic peaks and appearance of hydrolysis products in the mass spectrum [68] (Figure 1F). Furthermore, MALDI-TOF MS has shown promise in detecting carbapenem resistance, with proposed methods focusing on identifying bla_KPC_ carbapenemase genes carried on plasmids through spectral analysis [69]. Tools like MBT-ASTRA, which compare the area under the curves (AUCs) of bacterial spectra exposed to antibiotics versus those not exposed, have enabled rapid resistance profiling [70,71]. Another innovative approach, MBT-Resist, utilizes stable isotope labeling to detect shifts in bacterial mass spectra [72]. Moreover, Direct-on-target Microdroplet Growth Assay (DOT-MGA) offers a promising method for detecting antibiotic resistance directly on the MALDI-TOF MS target plate [73]. The detection of resistance-related peaks in MALDI-TOF MS provides early but limited information on antibiotic resistance. However, the development of new analytical algorithms, automation of procedures, and optimization of detection methods are expected to enhance and expand the clinical applications of MALDI-TOF MS in microbiological diagnostics.

Despite their widespread use, these classical methods have limitations, such as the need for bacterial culturing and their inability to detect all forms of resistance. As a result, advanced molecular techniques like genome sequencing and machine learning are increasingly integrated to complement traditional approaches. These technologies provide a more comprehensive understanding of both the genetic and phenotypic factors that contribute to antibiotic resistance in *E. coli*. 

## 3. Mechanisms of Antimicrobial Resistance in *E. coli*

*E. coli* antibiotic resistance can be mainly categorized into exogenous and endogenous mechanisms. The antibiotic resistance of *E. coli* mediated by exogenous genes primarily occurs through mechanisms like drug inactivation and target modification. Additionally, some antibiotics are expelled from the cell by efflux pumps, further contributing to resistance. Drug inactivation involves bacteria neutralizing antibiotics either by breaking them down or chemically modifying them. This process can occur through hydrolysis, where the antibiotic is destroyed, or by adding chemical groups that render the antibiotic ineffective. Target modifications, on the other hand, change the structure of the antibiotic’s target, preventing the antibiotic from binding effectively. This enables the target to function normally and allows the bacteria to develop resistance. In addition to acquiring external genes that modify targets, bacteria can also develop resistance through mutations in their own genes that change the target’s structure [74]. In addition to acquiring foreign resistance genes, certain endogenous genes can also confer antibiotic resistance to strains. For example, multidrug transporters in *E. coli* can remove a variety of harmful molecules from the cell, including antibiotics. Recent studies have also revealed that mutations in genes related to metabolic pathways can contribute to the development of antibiotic resistance in *E. coli*.

### 3.1. Drug Inactivation and Modification

**Figure 2:**
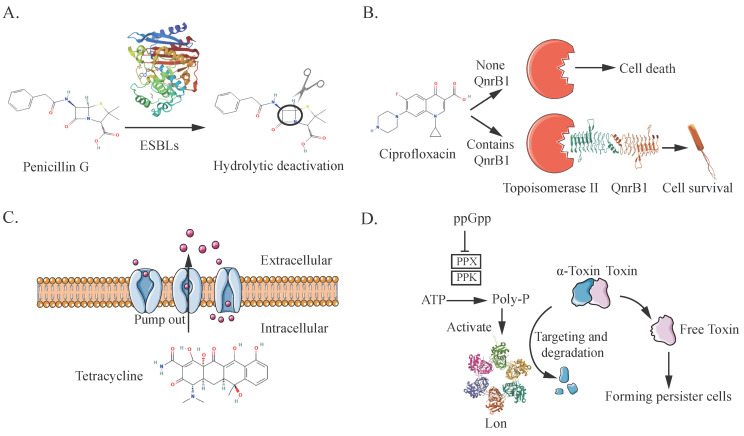
Common Mechanisms of AMR in *E. coli*. (**A**) Resistant strains arise through inactivation or modification of antibiotics. (**B**) Resistance also occurs by modifying the target’s structure or blocking antibiotic binding, allowing the target to function properly. (**C**) Efflux pumps enhance resistance by actively expelling antibiotics from the cell. (**D**) Persistence is achieved by forming dormant cells that can tolerate and survive antibiotic treatment.

The inactivation and modification of antibiotics enhance bacterial tolerance to these drugs. Key mechanisms include the hydrolysis of β-lactam antibiotics by β-lactamases and the inactivation of tetracycline through tetracycline hydroxylase, as illustrated in Figure 2A. Penicillinase, discovered in 1940, was the first enzyme identified that destroys penicillin by hydrolyzing the β-lactam ring, leading to resistance [75]. The Beta-lactamase Database (BLDB) [76] catalogs enzymes that degrade β-lactam antibiotics by hydrolyzing the amide bonds in the β-lactam ring, thereby promoting antibiotic resistance. Additionally, the BLDB provides tools for users to conduct BLAST searches of protein and nucleotide sequences, aiding in the investigation of the evolution of antibiotic resistance. As of 23 July 2024, the Beta-lactamase Database (BLDB) includes a total of 8273 enzymes, each characterized by various properties. BLDB categorizes these enzymes into four classes based on their amino acid sequences, substrate profiles, and inhibitor interactions: Class A includes penicillinases such as TEM, SHV, and CTX-M, which primarily hydrolyze penicillins, as well as KPC, a carbapenemase that also hydrolyzes penicillins and other β-lactams. Class B: Comprises metallo-β-lactamases (MBLs) such as NDM and VIM, which have broad substrate specificity but do not act on monobactams. Class C: Contains cephalosporinases like CMY and ADC, which preferentially act on cephalosporins. Class D: Known as oxacillinases (OXA), including OXA-48 and OXA-23, targeting oxacillin and similar penicillins [77,78]. Carbapenems, known for their broad antibacterial spectrum and strong activity, are typically resistant to β-lactamases and are crucial for treating serious infections. However, carbapenem-hydrolyzing enzymes like KPC, IMI, and GES from Class A, and OXA-48, OXA-23, and OXA-40 from Class D, along with IMP, VIM, and NDM from Class B, present significant resistance challenges. These β-lactamases are often transmitted via plasmids, as seen with the carbapenem-resistant bla_NDM-1_ gene in New Delhi [79] and the bla_CTX-M-15_ and bla_CTX-M-55_ genes in contaminated environments [80,81]. β-lactamase genes can also be associated with insertion sequences, such as ISAba1, which enhances the expression of cephalosporinases [57].

Tetracyclines are a class of broad-spectrum antibiotics that work by binding reversibly to the 30S subunit of bacterial ribosomes. This binding inhibits protein synthesis by preventing aminoacyl tRNA from attaching to the ribosomal A site, thereby blocking bacterial growth [82]. Tet (X) is a flavin-dependent monooxygenase that inactivates antibiotics through monohydroxylation and spontaneous non-enzymatic degradation. The Tet (X) family can mobilize on transposable elements, leading to high levels of tetracycline resistance [83,84]. Tigecycline is an important antibiotic used to treat severe infections caused by multidrug-resistant bacteria. Plasmid-mediated resistance genes tet (X3) and tet (X4) have been identified in humans, animals, and edible meats, conferring high levels of resistance to all tetracyclines, including tigecycline, minocycline, and omadacycline [85].

Another mechanism of antibiotic inactivation involves modifying the antibiotic molecule by adding chemical groups through enzyme activity. This modification hinders the antibiotic’s ability to bind to its target protein, leading to bacterial resistance [86]. Drug-modifying enzymes can transfer various chemical groups, such as phosphoric acid, acyl, ribose, and nucleotides, rendering bacteria resistant to antibiotics like aminoglycosides, macrolides, streptomycin, chloramphenicol, lincomycin, and rifamycin. Table 1 summarizes the enzymes that inactivate antibiotics by modifying functional groups, along with their mechanisms.

Aminoglycoside antibiotics, which contain numerous exposed hydroxyl and amide groups, are particularly susceptible to modification by enzymes such as acetyltransferases, phosphotransferases, and nucleotide transferases [87,88]. Macrolide antibiotics are rendered less effective when modified by enzymes such as phosphotransferases and esterases, which prevent them from properly binding to the bacterial 50S ribosomal subunit [89]. Streptomycin and chloramphenicol are also inactivated by acetyltransferases. Lincomycin antibiotics, which bind to the 23S rRNA of the 50S ribosomal subunit to disrupt peptidyl transferase reactions, can be inactivated by nucleotide transferases encoded by the *lnu* gene through the addition of a phosphoric acid-containing group [90]. Rifampicin (RIF) interacts with the β subunit of RNA polymerase, which is encoded by the *rpoB* gene, at a site deep within the DNA/RNA exit tunnel. RIF binds to RpoB through a series of hydrogen bonds with the naphthol ring hydroxyl groups and the hydroxyls on the naphthol ring at positions C21 and C23 of the ansa bridge, disrupting the elongating mRNA chain and causing premature transcription termination [91]. *E. coli* can inactivate rifampicin through ADP ribosyl transferase, glycosyl transferase, phosphotransferase, and monooxygenase [92,93]. ADP ribosyltransferases Arr catalyze ADP ribose from the cofactor NAD^+^ to the hydroxyl linked to C23 of the antibiotic, thereby sterically blocking productive interaction with RNA polymerase [94]. Similar to ADP ribosyltransferases, rifamycin glycosyltransferases (Rgt) modify the hydroxyl group at the C23 position of rifamycins by transferring a glucose moiety from the donor molecule UDP-glucose [95,96]. Rifampicin phosphotransferase phosphorylates and transfers the β-phosphate of ATP to the hydroxyl group on the naphthol ring at position C21 of the rifampicin ansa chain, hindering its interaction with RpoB and leading to the development of resistance in the strain [97,98]. Rifampicin monooxygenase Rox is a fad-dependent enzyme that hydroxylates the C2 position of the naphthoquinone core. This reaction leads to the cleavage of the C-N bond at C2 and the opening of the ansa chain, causing separation of the naphthyl portion from the cyclic aliphatic chain. This structural alteration disrupts the three-dimensional conformation of rifampicin, resulting in its inactivation [99,100,101,102].

**Table 1:** Enzymes that Inactivate Antibiotics by Modifying Functional Groups and Their Mechanisms.

Antibiotics	Antibiotic Binding Site	Enzymes	Mechanism
Aminoglycoside	Binding to the 16S rRNA of the 30S ribosomal subunit results in mRNA misreading and the production of non-functional, misfolded proteins [103].	Acetyltransferase	Transfers acetyl groups from acetyl-CoA, reducing the drug’s ability to interact with the ribosome.
		Phosphotransferase	Transfers phosphate groups from ATP.
		Nucleotide transferase	Transfers nucleotide triphosphate groups.
Macrolides	Inhibits bacterial protein synthesis by blocking peptidyl transferase activity in the 50S ribosomal subunit.	Phosphotransferase	
		Esterase	
Streptomycin	Prevents aminoacyl-tRNA from entering the A site of the 50S ribosomal subunit, hindering bacterial protein synthesis.	Acetyltransferase	Group A binds to the peptidyl transferase center, while group B binds to the peptide exit tunnel.
Chloramphenicol	Blocks transpeptidylase activity by preventing the binding of aminoacyl-tRNA to the 50S subunit, inhibiting the formation of new peptide chains.	Acetyltransferase	Transfer acetyl groups from coenzyme A to inhibit chloramphenicol binding to its ribosomal target.
Lincosamides	Binds to 23S rRNA in the 50S ribosomal subunit, inhibiting the peptidyl transferase reaction.	Nucleotide transferase [90]	Add a phosphoric acid group to the antibiotic.
Rifamycin	Binds to 23S rRNA in the 50S ribosomal subunit.	ADP ribosyltransferase [92]	Transfer ADP-ribose from NAD to the C23 hydroxyl group of the antibiotic.
		Glycosyltransferase [92]	Transfer glucose from UDP-glucose to partially modify the C23 hydroxyl group of the antibiotic.
		Phosphotransferase [92]	ATP-dependent dikinase transfers the β-phosphate of ATP to the C21 hydroxyl group of rifamycin’s ansa bridge.
		Monooxygenase [92]	Fad-dependent enzyme hydroxylates the naphthoquinone core and separates the naphthalene chain from the naphthalene base.

### 3.2. Target Modification Confers Antibiotic Resistance

Target modification can happen through modifying enzymes and proteins, as well as by mutations. Most antibiotics function by binding specifically to their molecular targets within bacteria. This binding can be blocked either by altering the structure of the target or by chemically modifying it. This allows the target to continue functioning normally and conferring resistance to the strain [104]. These modifications are often facilitated by foreign genes carried by plasmids. For example, quinolone antibiotics target topoisomerase II (DNA gyrase) (Figure 2B) and topoisomerase IV. Research has shown that plasmid-encoded quinolone resistance (*qnr*) genes can protect these targets by binding to them to make the strain resistant. For example, the plasmid QnrB1 encoding pentapeptide repeat protein (PRP) binds to the topoisomerase. It can protect the activity of the DNA gyrase enzyme [105]. To date, seven different qnr families—QnrA, QnrB, QnrC, QnrD, QnrE, QnrS, and QnrVC—have been identified as playing a role in protecting these targets [106].

Another mechanism of antibiotic resistance involves adding chemical groups to the drug target, preventing antibiotics from binding effectively. For example, resistance to aminoglycoside antibiotics can occur through methylation of ribosomal targets by 16S rRNA methyltransferases. This modification prevents antibiotics from binding to the 30S ribosome [107], such as *armA* gene [108] and *rmt* genes [109] found in *E. coli*.

A plasmid carrying the multidrug resistance gene *cfr* has been identified in *E. coli* and *S. aureus*. This gene encodes a methyltransferase that modifies the 23S rRNA at position A2503, resulting in resistance to a wide range of antibiotics, including lincoamides, streptogramin A, phenicols, pleuromutilins, 16-membered macrolides, and the oxazolidinone linezolid [110,111]. Polymyxin, a cyclic antimicrobial peptide with a long hydrophobic tail, targets lipopolysaccharide (LPS) in bacterial cell membranes, disrupting the hydrophobic interactions and causing outer membrane damage and bacterial death [112]. Bacterial resistance to polymyxin is mediated by phosphoethanolamine (PEA) transferases, which transfer PEA from phosphatidylethanolamine (PE) to lipid A’s phosphate group, altering the molecule’s charge through LPS modification [113,114]. In 2015, a new PEA transferase named Mcr was discovered, and ten *mcr* genes and their variants have been identified since then [115].

Another common mechanism of target modification involves altering the antibiotic’s target through mutations, preventing the antibiotic from binding effectively and thereby conferring resistance to the strain. These mutations often occur in the organism’s endogenous genes. For example, β-lactam antibiotics work by inhibiting penicillin-binding proteins (PBPs), and introducing four amino acid substitutions in *E. coli* PBP3 can make the strain resistant to multiple cephalosporins [116]. Similarly, resistance to quinolones can occur not only through the protection of topoisomerase by exogenous Qnr proteins but also due to mutations in the *gyrA*, which together lead to high levels of resistance [106]. Rifampicin exerts an inhibitory effect on RNA polymerase (RNAP), and mutations in RNAP can lead to resistance against rifampicin [27,117,118,119]. These mutations can alter the conformation of RNAP, affecting its ability to bind to antibiotics. For instance, rifampicin binds to the β subunit of RNAP, interfering with RNA synthesis, while mutations in the *rpoB* gene cause conformational changes in RNAP that reduce the inhibitory effect of the antibiotic [20,120].

### 3.3. Mechanisms of Drug Transport in AMR

*E. coli* can efflux antibiotics from the cell using efflux pumps, contributing to antibiotic resistance. Several multidrug transport systems have been identified within the transporter superfamily, including the ABC family, SMR family, RND family, and MFS family. These transport systems share similar specificity for various lipophilic planar molecules with molecular weights under 800 Da [121]. The ABC transporter family uses the energy from ATP binding and hydrolysis for transport and consists of two transmembrane domains (TMD) and two nucleotide-binding domains (NBD) [122,123]. On the other hand, the SMR, RND, and MFS families are secondary transporters that utilize the movement of protons along a transmembrane electrochemical gradient to provide energy for drug transport, without the need for ATP binding or hydrolysis.

The ABC (ATP-binding cassette) transporter family is present in a variety of organisms. By constructing *E. coli* strains lacking the primary efflux system AcrB (including Δ*acrB*, Δ*acrB*/Δ*yddA*, and Δ*yddA* mutants), it was found that *yddA*, derived from the *E. coli* K-12 strain, functions as a multidrug efflux gene that transports drugs through ATP hydrolysis [124].

The RND (Resistance-Nodulation Division) family, primarily represented by AcrB in *E. coli*, can expel various antibiotics such as minocycline and doxorubicin from the cell [125]. Since some antibiotic targets, like β-lactams, are located in the periplasmic space. Therefore, transporters like AcrB must expel these compounds from both the inner membrane and the periplasm [126]. AcrB functions as a complete membrane protein with a transmembrane domain (TMD) composed of 12 transmembrane alpha helices and a large periplasmic domain [127]. AcrB operates in conjunction with the membrane fusion protein AcrA and the outer membrane channel TolC, transporting drugs through the TolC pore across the periplasm and outer membrane (Figure 2C) [128,129,130]. The binding of the inner membrane protein–protein AcrZ to the AcrAB-TolC complex enhances the efflux of tetracycline, chloramphenicol, and puromycin [131,132]. Additionally, AcrD from the *E. coli* RND family can expel aminoglycosides from both the cytoplasm and periplasm [133], as well as remove bile acids, neobiotin, and fusidic acid [134].

The SMR family (Small Multidrug Resistance) is primarily represented by EmrE in *E. coli* and is known as the smallest multidrug transporter. EmrE functions as a dimer composed of four transmembrane alpha helices, with its 2 subunits aligned oppositely across the membrane [135,136]. EmrE expels aromatic cationic antibiotics via proton-coupled efflux, conferring resistance. Glu^14^, a membrane-embedded charged residue, plays a key role in substrate and ion recognition [137].

The MSF family (Major Facilitator Superfamily) in *E. coli* is represented mainly by EmrD, which has a broad substrate range [138]. EmrD is a compact protein consisting of 12 transmembrane α helices, organized into two layers: an outer layer and an inner layer, each made up of 6 α helices. The outer α helices of EmrD are similar to those found in the lactose permease enzyme LacY [139] and the glycerol-3-phosphate transporter GlpT [140]. However, the inner layer forms a larger cavity, allowing EmrD to accommodate a wider range of substrates compared to LacY and GlpT. EmrD primarily transports aromatic drugs.

The *mar* operon in *E. coli* regulates the expression of efflux pumps and porins, contributing to multidrug resistance. This regulation is mediated by transcription factors MarR and MarA, which activate genes involved in DNA repair and lipid transport [141]. MarA activates the transcription of genes involved in both drug efflux and membrane integrity, including those necessary for lipid trafficking and DNA repair. MarA activates genes such as *xseA*, which encodes a subunit of Exonuclease VII. This enzyme plays a crucial role in DNA repair. Effective DNA repair mechanisms reduce the accumulation of lethal DNA damage, allowing the bacterial cells to withstand higher concentrations of antibiotics that would typically lead to cell death. MarA also regulates genes in the *mlaFEDCB* operon, which is responsible for lipid transport in the outer membrane. By maintaining membrane integrity and asymmetry, the *mlaFEDCB* operon reduces the permeability of the outer membrane to hydrophilic antibiotics. This decrease in permeability restricts the entry of these drugs into bacterial cells, thereby increasing resistance. MarA enhances repair mechanisms and maintains membrane integrity, allowing bacteria to better survive antibiotic treatment. This highlights the complex interactions between different resistance mechanisms.

Quinolone resistance in *E. coli* is mediated by two major efflux pumps: QepA [142] and OqxAB [106]. The qepA gene is associated with a transposition element flanked by two copies of IS26. *E. coli* containing qepA exhibits significantly higher resistance levels to norfloxacin, ciprofloxacin, and enrofloxacin [142].

### 3.4. Metabolic Adaptations in Antimicrobial Resistance

The metabolic status of bacteria significantly influences their susceptibility to antibiotics, and adjusting this status can lead to increased antibiotic resistance in bacterial strains [143]. For example, *E. coli* can develop multidrug resistance by forming persister cells during growth. By altering the levels of secondary messenger nucleotides, such as ppGpp and pppGpp, bacterial stress responses can be modulated. Enhanced (p)ppGpp levels cause RpoS regulation, DNA replication, transcription, and translation, while also activating virulence and toxin–antitoxin modules [144].

HipA in *E. coli* is recognized as a key inducer of the persistence phenotype, which mutants allow for temporary survival under antibiotic stress like phosphomycin, cycloserine, and ampicillin [145]. HipA interacts with the transcriptional regulator HipB and the *hipBA* promoter, forming a toxin–antitoxin (TA) module that modulates the activity of HipA [146]. Single-cell analysis indicates that random overexpression of HipA significantly increases the formation of persistent cells in *E. coli*. As a serine protein kinase, HipA phosphorylates glutamyl-tRNA synthetase, inhibiting protein synthesis and leading cells into a dormant state. This dormancy is a critical aspect of the persistence phenomenon, enabling a subset of the population to survive antibiotic treatment. While HipA does not confer permanent multidrug resistance, its action facilitates survival during antibiotic exposure, as seen in chronic infections like urinary tract infections. For instance, the *HipA7* mutant, which carries mutations G22S and D291A, significantly enhances persister cell formation by 1000-fold [147]. Whole-genome sequencing revealed that the HipA allele P86L conferred persistence levels similar to those of the HipA7 mutant, significantly enhancing survival under antibiotic pressure. In a further study, 477 *E. coli* isolates from patients with urinary tract infections were screened, identifying 23 patients with *hipA7* mutants (all with G22S and D291A replacements) and one with the HipA (P86L) mutation. HipA7 promotes the establishment of a persistent state by inducing the synthesis of (p)ppGpp, promoting a rapid transition of cells to a dormant state after drug exposure (Figure 2D). This mechanism enables a greater proportion of *E. coli* to enter a persistent state, thereby improving survival during antibiotic treatment.

In *E. coli*, Obg (also known as *ObgE*, *CgtA*, *YhbZ*) plays a crucial role in regulating cell persistence. Obg is a conserved P-loop GTPase involved in translation and DNA replication, which enables persistence by responding to (p)ppGpp and regulating the type I toxin–antitoxin module *hokB-sokB*. Increased levels of Obg promote the expression of HokB, reducing membrane potential and driving cells into dormancy [148].

Changes in carbon sources stimulate the formation of persistent cells in *E. coli* that are resistant to fluoroquinolone antibiotics. Specifically, using a glucose-fumarate dual carbon source, instead of glucose alone, led to an increase in *ofloxacin* persistence when glucose was depleted. This depletion activates (p)ppGpp and inhibits DNA gyrase, resulting in resistance to fluoroquinolones [149]. (p)ppGpp inhibits the transcription of genes necessary for growth while upregulating stress response genes. This can lead to a reduction in the synthesis of proteins required for DNA replication and gyrase activity. Additionally, high levels of (p)ppGpp may result in decreased ATP availability, which is critical for the activity of DNA gyrase. The accumulation of (p)ppGpp alters the overall metabolic state of the cell. This change leads to a decline in the resources needed for enzyme activity. Therefore, the effect on DNA gyrase is indirect rather than a direct interaction.

Single metabolic stresses can induce specific persistence in cells. For example, RelA is required for ampicillin-tolerant persistence in *E. coli*, but this process is disrupted by the absence of *clpA*, *ssrA*, or *smpB*. Common mediators like ppGpp, DksA, SsrA, and SmpB contribute to both ofloxacin and ampicillin persistence, while ClpA is specific to ampicillin and nucleoid-associated proteins to ofloxacin [150].

Mutations in Complex I of the *E. coli* respiratory chain promote cellular persistence. In laboratory evolution studies examining genomic changes in *E. coli* exposed to antibiotics, an increase in mutations in the *nuo* operator region encoding Complex I was observed. These mutations were primarily found in the transmembrane domain, leading to damage in the proton pump, which intensified cytoplasmic acidification and contributed to high persistence. Cells experiencing nutrient deprivation or transport stagnation often disrupt the metabolic balance, further acidifying the cytoplasm. In wild-type strains, this disturbance can be mitigated by activating the RpoS regulator, but in *nuo* mutants, acidification prevents protein synthesis, enhancing persistence [14].

The cell wall is the target of β-lactam antibiotics. In *E. coli*, penicillin-binding proteins (PBPs) catalyze D,D-transpeptidase activity to cross-link peptidoglycan, forming the cell wall. However, L,D-transpeptidase YcbB can bypass this mechanism, conferring resistance to β-lactam antibiotics. YcbB, along with PBP5 (D,D-carboxypeptidase) and PBP1b, plays a role in this D,D-transpeptidase bypass. (p)ppGpp acts as a signaling molecule that regulates various cellular processes, including stress responses and antibiotic resistance mechanisms. When (p)ppGpp levels rise, it triggers a series of reactions that enhance the cell’s ability to adapt to stress. Elevated (p)ppGpp levels can upregulate the expression of genes encoding YcbB and other proteins involved in peptidoglycan synthesis, including PBP5 and PBP1b. PBP5 is a D,D-carboxypeptidase that modifies the peptide side chains of peptidoglycan precursors, while PBP1b possesses both glycosyltransferase and transpeptidase activities. Together, they create substrates suitable for YcbB-mediated crosslinking. Under high (p)ppGpp conditions, YcbB interacts more effectively with PBP5 and PBP1b, facilitated by structural adaptations or post-translational modifications that enhance these protein interactions. The binding of PBP5 produces tetrapeptide donor substrates through its carboxypeptidase activity, which is crucial for YcbB’s L,D-transpeptidase activity, allowing the cell to bypass the normally β-lactam-inhibited D,D-transpeptidase activity. The increased interactions among YcbB, PBP5, and PBP1b enable *E. coli* to maintain peptidoglycan crosslinking even in the presence of β-lactam antibiotics, thereby contributing to increased resistance [151,152].

Research on the role of metabolism in antimicrobial resistance has shown that strongly metabolically dependent antibiotics are more likely to induce resistance. In contrast, weakly metabolite-dependent antibiotics typically do not lead to resistance. Additionally, the deletion of the *nhaA* gene, which disrupts cellular homeostasis under alkaline conditions, may enhance stress responses and contribute to ampicillin resistance in *E. coli* [153]. This suggests that targeting the metabolic dependence of antibiotics could provide new strategies to reduce resistance.

### 3.5. Resistance Mechanisms to Recently Approved Antibiotics in E. coli

Although the recently approved antibiotics, such as ceftazidime-avibactam, ceftolozane-tazobactam, meropenem-vaborbactam, and plazomicin, have only been in clinical use for a few years, emerging resistance mechanisms have already been reported. These mechanisms highlight the dynamic nature of bacterial adaptation and underscore the need for ongoing surveillance [154,155,156].

Ceftazidime-avibactam (CAZ-AVI) is a novel combination of the broad-spectrum cephalosporin ceftazidime (CAZ) and the β-lactamase inhibitor avibactam (AVI), designed to overcome resistance mediated by certain β-lactamases. CAZ-AVI is highly effective against Class A, C, and D β-lactamases, which inactivate antibiotics by hydrolysis, modify the antibiotic target, or upregulate efflux pumps [157,158]. In *E. coli*, resistance to CAZ-AVI can be attributed to several factors. Notably, KPC variants, such as KPC-2, with mutations in the Ω-loop (e.g., Asp179Asn), extend the half-life of the covalent enzyme-β-lactam intermediate, allowing prolonged “capture” of β-lactams. This results in a reduced dissociation rate of the enzyme-β-lactam complex, enabling the β-lactams to bind more tightly to the enzyme than avibactam, ultimately leading to resistance [159,160]. Additionally, mutations in the CTX-M-15 enzyme, such as the Asp182Tyr substitution, also contribute to CAZ-AVI resistance by altering the enzyme’s ability to hydrolyze the drug [161]. Furthermore, mutations in the target protein, PBP3, such as the insertion of four amino acids (Thr-Ile-Pro-Tyr), can increase the MIC of CAZ-AVI from ≤1 mg/L to 2–8 mg/L [162].

Ceftolozane-tazobactam (C/T) is another important β-lactam/β-lactamase inhibitor combination approved for use against multidrug-resistant *E. coli*. Resistance mechanisms in *E. coli* to C/T include PBP3 alterations, where mutations in the penicillin-binding proteins (PBPs) decrease the binding affinity of ceftolozane [163,164]. Additionally, β-lactamase overproduction, particularly the upregulation of AmpC β-lactamases, and mutations in efflux pumps and porin channels are known to play a role in resistance to C/T. The AmpC omega loop has been identified as a key region involved in substrate binding, and mutations in this region can lead to resistance to ceftolozane [165].

Meropenem-vaborbactam (MER-VAB) is a carbapenem and β-lactamase inhibitor combination approved in 2017, primarily used for treating carbapenem-resistant bacteria [166]. It is effective in inhibiting Class A and Class C β-lactamases, especially the KPC enzymes. However, carbapenem-resistant Enterobacteriaceae (CPE) that produce Class D or Class B carbapenemases are generally resistant to MER-VAB. Vaborbactam inhibits KPC, and its structure differs from other β-lactamase inhibitors like avibactam and relebactam. Resistance to MER-VAB in *E. coli* can be mediated by the presence of metal-β-lactamase genes such as bla_NDM-1_ and bla_NDM-5_, as well as the bla_OXA-48_ gene [167]. Additionally, some clinical isolates of *E. coli* resistant to MER-VAB have been found to carry resistance mechanisms involving β-lactamase genes (such as CTX and TEM) and mutations in outer membrane protein genes (*ompC* and *ompF*) [166]. In KPC-producing *Enterobacteriaceae*, the main mechanism of resistance to MER-VAB involves mutations in porins, which impair membrane permeability. This results in overexpression of β-lactamases and an increase in efflux pump activity, further contributing to resistance [156].

Plazomicin, a novel aminoglycoside approved in 2018, is used for treating urinary tract infections caused by multidrug-resistant *E. coli*. Resistance to plazomicin in *E. coli* is mediated by drug modification and target alterations. One known resistance mechanism is the acetylation of plazomicin at the 2′ position by AAC(2′)-Ia, an enzyme that methylates the G1405 position on the ribosomal RNA, thus decreasing the drug’s binding affinity and efficacy [168].

In conclusion, while recently approved antibiotics like CAZ-AVI, C/T, MER-VAB, and plazomicin represent promising tools in the fight against multidrug-resistant *E. coli*, bacterial resistance to these agents is already emerging through various mechanisms. These include β-lactamase mutations, target protein alterations, and changes in membrane permeability and efflux pump expression. These findings highlight the importance of continuous monitoring and the development of new treatment strategies to address the growing challenge of antimicrobial resistance.

## 4. The Impact of AMR on *E. coli* and Prevention Strategies

### 4.1. The Impact of AMR on E. coli

AMR significantly impacts the pathogenicity of *E. coli*, complicating treatment and increasing the risk of severe infections. *E. coli* is a common bacterium found in the intestines of humans and animals. While most strains are harmless, some can cause serious infections, including urinary tract infections, bloodstream infections, and gastrointestinal diseases. In recent years, there has been a marked increase in the prevalence of resistant *E. coli* strains [1]. The emergence of extended-spectrum beta-lactamase (ESBL)-producing *E. coli*, which are resistant to commonly used antibiotics such as penicillins and cephalosporins, is particularly concerning. Additionally, in certain regions, *E. coli* resistance rates to fluoroquinolones and other critical antibiotics have exceeded 50%. This not only complicates treatment, leading to increased hospitalization and healthcare burdens but also results in higher medical costs and increased mortality rates [169]. An analysis of the global burden of bacterial resistance from 1990 to 2021 indicates that AMR-related mortality is more pronounced in older populations and predicts that demographic changes will significantly affect the burden of AMR [170]. Environmental factors also play a crucial role in the dissemination of antimicrobial resistance; *E. coli* can be found in water sources, runoff from animal production systems, and contaminated food, serving as a vector for resistance genes [4,171]. The use of antibiotics in livestock can promote the development of resistant strains, which may be transmitted to humans through the food supply. Therefore, it is essential to strengthen regulations on antibiotic use in animal production to enhance food safety.

### 4.2. Emerging Approaches to Combat Antimicrobial Resistance

Antimicrobial resistance (AMR) poses a significant threat to the health of both humans and animals worldwide. To mitigate the risks associated with the emergence of antibiotic-resistant genes in the environment, certain measures need to be implemented, such as monitoring and risk assessment to identify critical control points [172]. Monitoring AMR dynamics from a health perspective is crucial. Using *E. coli* as an indicator for monitoring AMR occurrences and levels in the environment, including wildlife, can reveal both the anthropogenic impacts on antibiotic resistance and the transmission dynamics between human and animal populations [173]. In controlling the spread of AMR, phage therapy has been successfully applied to infectious diseases caused by superbugs. CRISPR-Cas technology allows for precise editing of resistance genes at the genetic level, demonstrating high accuracy and flexibility [174]. For example, targeting multidrug-resistant *Klebsiella pneumoniae*, a native CRISPR-Cas3 system was constructed to specifically target the IncFII plasmids responsible for the widespread dissemination of resistant strains, effectively eliminating IncFII resistance plasmids and restoring antibiotic sensitivity [175]. Moreover, by utilizing a host-compatible IncQ1 plasmid, a delivery vector called pQ-mini was created to eliminate resistance plasmids or genes in resistant strains such as *E. coli* CSZ4, *Salmonella* Typhimurium 15E556, and *Klebsiella pneumoniae* 43K [176]. Compared to plasmid vectors, phage vectors possess robust capabilities to infect host bacteria and can carry larger DNA fragments. By identifying conserved CRISPR/Cas9 target sequences from over 1000 *E. coli* ESBL mutants, phages have been used as delivery systems for Cas9 proteins and crRNA to inactivate β-lactamase gene mutations in *E. coli*, thereby restoring sensitivity to β-lactam antibiotics [177]. Research has found that a combination of four complementary phages, SNIPR001, shows good tolerance in both mouse and pig models, significantly reducing *E. coli* loads in the intestines compared to individual components. SNIPR001, a novel precise antibiotic that combines four CRISPR-Cas armed phages, has been developed to prevent bacteremia in patients with hematological malignancies undergoing chemotherapy [178]. Additionally, by covalently modifying proteins with cationic polymers and subsequently complexing them with single guide RNAs targeting antibiotic resistance genes, a nanoscale complex was successfully developed to target the methicillin resistance gene mecA in methicillin-resistant *S. aureus* [179]. IncHI plasmids, prevalent in *Enterobacteriaceae*, carry multiple resistance genes and encode high-molecular-weight proteins with bacterial Ig-like (Big) domains that are typically found on the bacterial surface, such as on flagella and pili. Developing nanobodies based on these proteins has been shown to interfere with the conjugative transfer of IncHI plasmids [180]. These approaches contribute to controlling the spread of AMR. However, it is equally important to strengthen the regulation of antibiotic use and establish standardized protocols at all levels to fundamentally address the issue of AMR transmission.

## 5. Conclusions

Antimicrobial resistance has become a major global health threat, with *E. coli* causing the most pathogen-related deaths in 2019. Researchers have used a variety of methods to research the mechanisms of antimicrobial resistance, such as machine learning for resistance pattern identification, laboratory evolution to generate resistant mutants, the construction of mutant libraries for deeper insights into resistance mechanisms, and genome sequencing for analyzing mutant strains. These methods have laid a foundation for developing new antibiotics and understanding how resistance emerges, guiding future research and drug development.

*E. coli* has been shown to acquire antimicrobial resistance through several mechanisms. These include inactivating or chemically modifying antibiotics, altering drug targets to prevent effective binding, expelling antibiotics via efflux pumps, and adjusting metabolism to withstand antibiotic stress. Additionally, segment duplications have been identified as a mechanism that confers resistance. While clinical data show that segment duplication can make pathogens like *E. coli* resistant, research in this area has been limited to isolated cases. Most studies focus on single-gene mutations or knockouts, and large-scale studies on segment duplication’s role in resistance remain unexplored.

Antimicrobial resistance (AMR) poses a significant threat to human and animal health, particularly affecting the pathogenicity of *E. coli*. The rise of resistant *E. coli* strains complicates treatment and increases the risk of severe infections. Effective strategies to combat AMR include monitoring resistance dynamics, employing innovative therapies such as phage therapy and CRISPR-Cas technology, and enforcing stricter regulations on antibiotic use in agriculture. Addressing these challenges is essential to mitigate the impact of AMR and ensure the effectiveness of existing antibiotics in treating infections. In recent years, the rapid development of synthetic biology, machine learning, and artificial intelligence has introduced new methods and mechanisms for studying antimicrobial resistance.
